# Altered Functional Connectivity between Emotional and Cognitive Resting State Networks in Euthymic Bipolar I Disorder Patients

**DOI:** 10.1371/journal.pone.0107829

**Published:** 2014-10-24

**Authors:** Giannis Lois, Julia Linke, Michèle Wessa

**Affiliations:** Department of Clinical Psychology and Neuropsychology, Institute of Psychology, Johannes Gutenberg-University Mainz, Mainz, Germany; University of Wuerzburg, Germany

## Abstract

Bipolar disorder is characterized by a functional imbalance between hyperactive ventral/limbic areas and hypoactive dorsal/cognitive brain regions potentially contributing to affective and cognitive symptoms. Resting-state studies in bipolar disorder have identified abnormal functional connectivity between these brain regions. However, most of these studies used a seed-based approach, thus restricting the number of regions that were analyzed. Using data-driven approaches, researchers identified resting state networks whose spatial maps overlap with frontolimbic areas such as the default mode network, the frontoparietal networks, the salient network, and the meso/paralimbic network. These networks are specifically engaged during affective and cognitive tasks and preliminary evidence suggests that functional connectivity within and between some of these networks is impaired in bipolar disorder. The present study used independent component analysis and functional network connectivity approaches to investigate functional connectivity within and between these resting state networks in bipolar disorder. We compared 30 euthymic bipolar I disorder patients and 35 age- and gender-matched healthy controls. Inter-network connectivity analysis revealed increased functional connectivity between the meso/paralimbic and the right frontoparietal network in bipolar disorder. This abnormal connectivity pattern did not correlate with variables related to the clinical course of the disease. The present finding may reflect abnormal integration of affective and cognitive information in ventral-emotional and dorsal-cognitive networks in euthymic bipolar patients. Furthermore, the results provide novel insights into the role of the meso/paralimbic network in bipolar disorder.

## Introduction

Bipolar disorder (BD) is a heterogeneous disease characterized by acute dysfunctional mood states, alternating between mania (BD-I) or hypomania (BD-II) and depression, and related to dysfunctional emotion generation and regulation [1]. Further, BD patients suffer from cognitive impairments such as impaired verbal memory, deficits in executive functions and attentional deficits which are also present during remission [2], [3].

It has been proposed that the neural mechanisms underlying dysfunctional emotion regulation as well as cognitive impairments in BD are related to hypoactive ventral prefrontal areas that exert diminished top-down control on limbic brain structures resulting in hyperactivity of these structures [1], [4]–[7]. This disturbed frontolimbic functional connectivity (FC) has been shown during cognitive tasks [8], emotional tasks [9]–[11], and during resting state [12]–[16] in both symptomatic and euthymic BD patients.

Two main approaches to investigate functional brain connectivity are the seed-based analysis (SBA) and independent component analysis (ICA). SBA is a hypothesis-driven approach that correlates the time-series of the blood-oxygen-level-dependent (BOLD) signal of one brain region “seed” with the time-series of all other brain regions, resulting in a map that defines the functional connections of the predefined brain region. In contrast, ICA is a data-driven approach that identifies temporally coherent patterns of BOLD signal that are maximally independent from each other. ICA takes into account the BOLD signal from the whole brain to generate functional maps of different brain networks [17].

Studies that employed ICA to investigate functional connectivity during resting state were able to delineate a dozen of resting state networks (RSNs) that are consistent across subjects and sessions [18], [19] and show high concordance with measures of structural connectivity [20]. Additionally, the recently developed functional network connectivity (FNC) technique provides the possibility to quantify functional interactions between RSNs.

Interestingly, some of the identified RSNs are engaged during tasks that target affective and cognitive processes [17], [21]. For instance, the meso/paralimbic network (MPN) [22], [23] or else described as the medial temporal lobe network [24], composed of amygdala, hippocampus, parahippocampal gyrus, and temporal poles, is implicated in processing of emotional information and interoceptive awarenenss [21], [24], whereas the left and right frontoparietal networks (FPNs) are implicated in cognitive control and attention (right FPN) or language processes and working memory (left FPN) [17], [21] and are comprised of lateral prefrontal regions and inferior parietal cortex [25], [26]. Moreover, the salient network (SN) involved in the detection of salient stimuli [27] is mainly composed of the anterior insula and the anterior cingulate cortex [28]. The SN and the FPNs are broadly engaged during a wide range of cognitively demanding tasks [28]. In contrast to these networks, the default mode network (DMN), which is comprised of posterior cingulate cortex/precuneus, ventro-medial prefrontal cortex (vmPFC) and bilateral angular gyri, is strongly associated with self-referential activities and is deactivated during tasks that are directed towards external stimuli [29], [30]. Despite the differences between these RSNs, recent findings showed that the SN, the DMN and the FPNs functionally interact with each other [28], [31] and that deficits in engagement and disengagement of these 3 networks may be relevant to cognitive and affective dysfunctions [27], [32]. Taking into account the association of these RSNs with affective and cognitive functions known to be impaired in BD and given the overlap between the spatial maps of these RSNs and frontolimbic areas that are affected in BD, it can be argued that patterns of functional connectivity within or between these networks may be impaired in BD.

The majority of RS-fMRI studies in BD demonstrated abnormal ventral PFC connectivity with amygdala [14], [33] or other subcortical areas (i.e. thalamus, striatum) [12] using SBA. To date, only 4 RS-fMRI studies have employed ICA and investigated within or between-networks connectivity in adult BD-I patients. One study focused on the DMN and reported reduced engagement of the medial PFC within the DMN in manic BD patients [34]. Another RS-fMRI study compared within-network functional connectivity between psychotic BD patients, schizophrenia patients, their respective first degree relatives and healthy subjects using the ICA as a tool to explore potential psychosis endophenotypes [22]. They identified networks that may be implicated in BD (e.g. DMN, MPN, SN and FPNs) and they reported aberrant connectivity in psychotic BD patients within the posterior DMN and the MPN. These abnormalities were shared with schizophrenia patients but not BD relatives suggesting that these findings may be related to the common psychotic symptoms between the two clinical groups. Using a largely overlapping sample, the same research group investigated inter-networks connectivity in BD [23]. They found increased connectivity between the MPN and the SN in psychotic BD patients. This increased connectivity uniquely distinguished this group from healthy BD relatives and schizophrenia patients and correlated positively with the negative mood symptoms of BD patients [23]. Finally, a recent RS-fMRI study investigating both within- and between-network connectivity in BD and borderline personality disorder patients revealed aberrant connectivity patterns in BD between networks implicated in self-referential processing [35]. However, this study did not examine subcortical networks (i.e. MPN).

Given the sparse empirical evidence of RS-fMRI studies that employed a data-driven approach in BD, the present study sought to investigate functional connectivity within and between RSNs in BD using ICA. Our study extends prior work that used similar methodology by examining euthymic BD patients with minimum residual mood symptoms and no current psychotic symptoms. We focused on the DMN, the FPNs (i.e. right and left), the SN, and the MPN as these networks or brain regions within these networks have been previously implicated in BD [5], [22], [23], [34], [36], [37]. Based on the neurobiological model of BD and previous findings from RS-fMRI studies in BD, we expected to find altered functional connectivity within and between these RSNs that may reflect the underlying pathophysiology of the disorder. To further investigate whether abnormal functional connectivity patterns are affected by the clinical course of the disease, we examined the relationship between functional connectivity measures and some aspects of the history of the disease (i.e. onset of illness, time in remission, number of manic and depressive episodes, and history of psychotic symptoms) and medication. We focused on these variables as they describe sufficiently the clinical course of BD and some of them seem to correlate with behavioral variables [38], [39] and with brain activation and connectivity patterns [5], [13] within euthymic BD patients.

## Materials and Methods

### Ethical statement

The ethics committee of the Medical Faculty Mannheim of the University Heidelberg approved the study and all participants gave written informed consent before study participation. Participants/patients having legal guardians or caretakers were excluded from study participation. Only participants/patients, whose capacity to consent was not compromised were investigated in the present study. The capacity to consent was evaluated by senior clinicians as part of the diagnostic interview as well as by cognitive testing (forward and backward digit-span task, Culture Fair Intelligence Test (CFT-20)), which was in the normal range for all participants. All potential participants who declined to participate or otherwise did not participate were not disadvantaged in any other way by not participating in the study. The study has been conducted according to the principles expressed in the Declaration of Helsinki.

### Participants

We assessed 30 patients with bipolar I disorder (BD) who were recruited through epidemiological studies at the Central Institute of Mental Health in Mannheim, Germany, or local support groups. Thirty five healthy controls (HC) were recruited from the registry office of the city of Mannheim. Demographic differences between groups were not statistically significant (Table 1). Exclusion criteria for all participants were age under 18, neurological disorder or head trauma with unconsciousness, and common MRI exclusion criteria. Patients were also excluded if they fulfilled criteria of another Axis-I mental disorder as defined by the DSM-IV within the last 6 months and if they had lifetime diagnosis of rapid cycling, schizoaffective disorder or schizophrenia. To increase ecological validity of the patient sample, patients with lifetime (not current) Axis-I disorders were not excluded. More specifically, 7 patients met the criteria for lifetime history of substance abuse or dependence, and 3 patients met the criteria for lifetime anxiety and eating disorders (i.e. agoraphobia, social anxiety disorder, and bulimia). HCs were excluded if they fulfilled the criteria for any lifetime or current DSM-IV axis I mental disorder or took any psychotropic medication. The ethics committee of the Medical Faculty of Mannheim of the University Heidelberg approved the study and all participants gave written informed consent before study participation.

**Table 1:** Demographic and Clinical Characteristics of Participants.

Characteristics	BD (N = 30)	HC (N = 35)	Statistics	p-value
Gender (Female/Male)	17/13	20/15	χ^2^ = 0.34	0.853
Age, years, mean (SD)	40.83 (9.43)	41.94 (8.36)	T(65) = 0.5	0.62
Years of education, mean (SD)	14.97 (2.58)	14.89 (2.31)	T(65) = 0.6	0.54
**Current Symptoms, [range], mean (SD)**				
YMRS^a^ Score	[0–5] 1.3 (1.6)	[0–2] 0.11 (0.40)	T(65) = 3.9	0.00
BDI^b^ Score	[0–20] 6.7 (5.94)	[0–12] 1.26 (2.44)	T(65) = 5.5	0.00
HAM-D^c^ Score	[0–5] 0.83 (1.17)	[0–2] 0.14 (0.43)	T(65) = 3.2	0.02
**History of illness**				
No. of past depressive episodes, mean (SD)	3.63 (3.12)			
No. of past manic episodes, mean (SD)	2.66 (2.48)			
Age at illness onset, years, mean (SD)	23.73 (9.51)			
Time in remission, years, mean (SD)	3.08 (3.44)			
Medication load, mean (SD)	3.10 (2.35)			
History of psychotic symptoms (Yes/No)	13/17			

a YMRS = Young Mania Rating Scale,

b BDI = Beck Depression Inventory,

c HAM-D = Hamilton Depression Rating Scale.

### Diagnostic assessment

Main and comorbid diagnoses (for patients) and exclusion criteria (for HCs) were evaluated based on the Structured Clinical Interview for DSM-IV Axis I Disorders. The Young Mania Rating Scale, the Hamilton Rating Scale for Depression (HAM-D), and the Beck Depression Inventory (BDI) were administered to assess residual mood symptoms. Based on the clinical interview, patients were euthymic for at least two months before testing and had minimum residual symptoms (Table 1).

Variables describing the clinical course of the disease such as the number of past depressive and manic episodes, the age at illness onset, the time in remission, and the presence of psychotic symptoms were acquired for every patient. We also verified that the medication status of all patients had been stable during the past 6 months. Among the patients 4 did not take any medication, 25 patients took mood stabilizers (lithium: n = 6, valproate: n = 11, lamotrigin: n = 6, carbamazepin: n = 2), 11 patients took antidepressants (venlaflaxin: n = 3, citalopram: n = 2, sertralin: n = 2, duloxetin: n = 1, clomipramin: n = 1, mirtazapin: n = 1, opipramol: n = 1) and 11 patients took antipsychotic medication (quetiapin: n = 7, perazin: n = 1, risperidon: n = 1, olanzapin: n = 1, aripiprazol: n = 1) at the time of scanning. In accordance to previous studies [11], we coded the number and dosage of each medication and calculated a composite measure of total medication load. For antidepressants and mood stabilizers, we categorized each medication into low-dose (1 or 2 levels) or high-dose (3 or 4 levels) groupings as previously performed [40]. We added a no-dose subtype for those not taking these medications. Using a similar procedure, we estimated the clinical equivalency of the antipsychotic medication to Chlorpromazine dose equivalent, coding them as 0 (no medication), 1 (equal to or below the chlorpromazine dose equivalent), or 2 (above the chlorpromazine dose equivalent) [41]. A composite measure of medication load was generated by summing all individual medication codes for the two categories for each individual participant. The demographic and clinical characteristics of the two groups of participants are shown in Table 1.

### Data Acquisition

Data were acquired on a 3-T whole body scanner (Magnetom Trio, Siemens Medical Solutions, Erlangen, Germany). We conducted one high-resolution T1-weighted 3 dimensional MRI sequence (slice thickness = 1.1 mm, field of view = 256×240×176 mm^3^, TR = 2.3 s, TE = 2.98 ms). We acquired 40 gradient-echo T2*-weighted slices (slice thickness = 2.3 mm) per volume with the following parameters: TR = 2.7 s, flip angle = 90°, TE = 27 ms, field of view = 220 mm^2^, matrix = 96×96, voxel size = 2.3 mm×2.3 mm×2.3 mm. During acquisition of resting-state, participants were instructed to lie still with their eyes closed and not to fall asleep for 5 minutes. 120 whole-brain volumes were acquired with the initial 4 images being discarded to allow for T2 stabilization effects.

### Data Analysis

#### Pre-Processing

Imaging data were preprocessed according to standard procedures using SPM8 (http://www.fil.ion.ucl.ac.uk/spm). Preprocessing involved realignment to the mean image of each run, slice-timing, and normalization into Montreal Neurological Institute standardized space (http://www.mni.mcgill.ca). During normalization, the images were resampled every 3 mm using sinc interpolation and smoothed with a 9×9×9 mm Gaussian kernel to decrease spatial noise. One BD patient and one HC were excluded from the initial sample (i.e. 31 BD patients and 36 HC) due to excessive movement during the fMRI scans (i.e. more than 3 mm translation or 3 degrees rotation and 2 mm or degrees in the first derivatives of motion parameters). In order to exclude the possible confound of different motion artefacts in the compared groups, we additionally estimated the maximum absolute displacement of each brain volume as compared to the previous volume from the translation parameters in the x (left/right), y (anterior/posterior), and z (superior/inferior) directions [42]. No group differences were present in the maximum motion (t = 1,32, df = 65, p = 0.19). This variable was used as nuisance regressor in the group comparisons.

#### Group-ICA

The Group ICA of fMRI toolbox (GIFT; version 1.3i; http://icatb.sourceforge.net) [43] was used to carry out a group ICA in the preprocessed and normalized data. Using the minimum description length criteria to determine the number of independent components (ICs), we were not able to identify all the RSNs of interest. It has been shown that increasing the number of ICs yields refined components that correspond to known anatomical and functional segmentations [17], [44], [45]. Following previous procedures [44], [45], we gradually increased the number of ICs to 40 until all networks of interest were identified. An initial data reduction step used principal component analysis on the subject-specific data and was followed by an IC estimation that produced 40 time courses and spatial maps with the Infomax algorithm [46]. This algorithm was repeated 20 times in Icasso, each time with different initial conditions. Resulting components of different runs were clustered to estimate the reliability of the decomposition [47]. The index *Iq*, which ranges from 0 to 1, reflects the difference between intra-cluster and extra-cluster similarity [47]. Each of the 40 components had a cluster quality index greater than 0.8. Group-level spatial maps were estimated as the centrotypes of component clusters to reduce sensitivity to initial algorithm parameters. These components were identified in the group of 65 subjects without differentiating between HCs and BD patients to ensure that the same components are identified in each group. Group-level ICs (both spatial maps and time courses) were then back-reconstructed for each subject using the GIGA method [43], [48]. Therefore, the subject-specific time course of each component represented a pattern of synchronized brain activity, whose coherency pattern across voxels was represented in the associated spatial map. Component intensity values were then z-scaled to provide normalized scores across subjects.

Based on previous ICA studies and to validate our results, we repeated the Group ICA analysis with 75 ICs components; this high model order yield refined components that correspond to known anatomical and functional segmentation (see Analysis S1).

#### Identifying Valid RSNs

**Figure 1:**
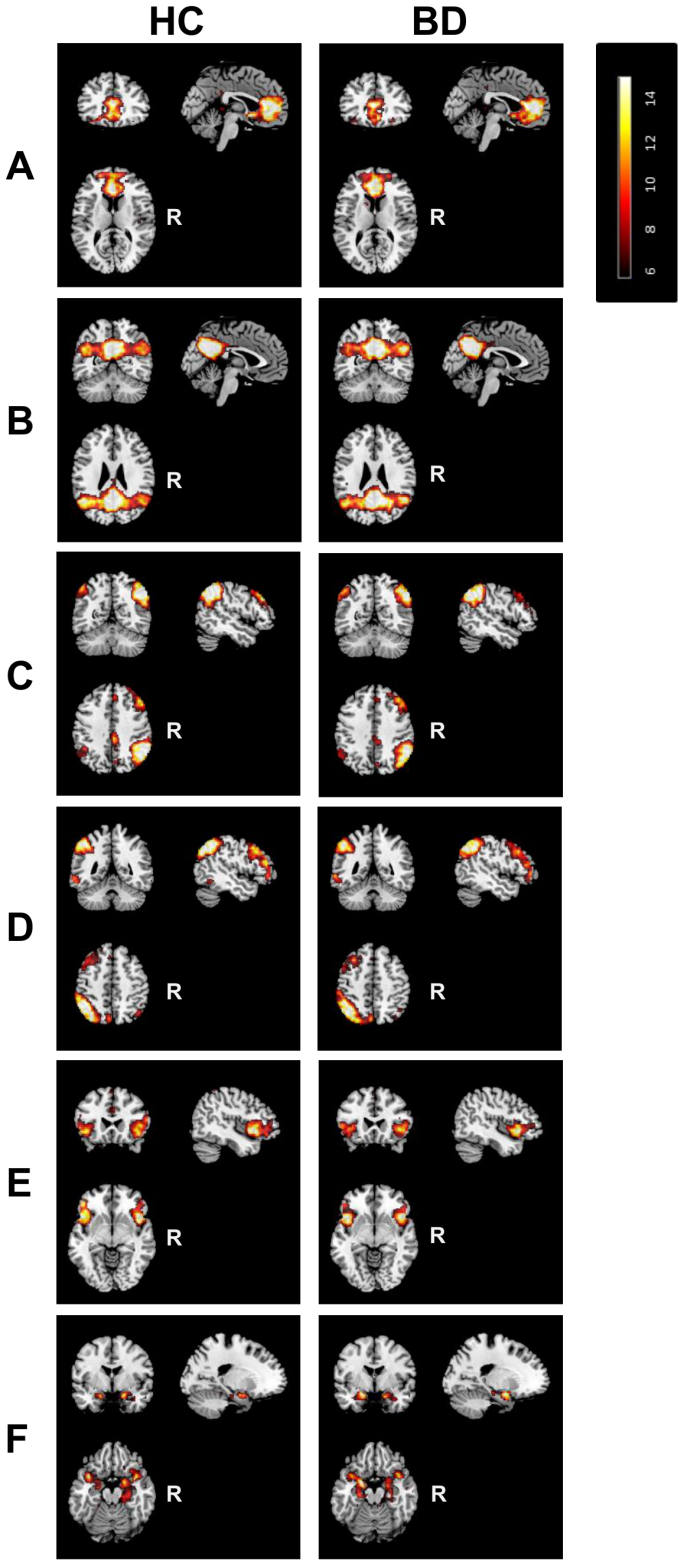
Resting state networks of interest. Illustration of one-sample-t-test maps of the anterior and posterior default mode network, right and left frontoparietal network, the salience network, and the meso/paralimbic network identified in the control (left column) and patient (right column) group. Maps are thresholded at P<0.05 (whole-brain FWE corrected). R, Right.

To identify valid RSNs, we first estimated the voxel-wise spatial overlap of the ICs with SPM's standard tissue-type probability maps for gray matter, white matter and cerebrospinal fluid (http://imaging.mrc-cbu.cam.ac.uk/imaging/Templates) using Pearson's correlation (Table S1). Subsequently, group-level ICs were spatially sorted in GIFT toolbox using templates of the RSNs of interest (i.e. DMN, right and left FPN, SN, MPN; Table S1). These templates were based on whole-brain task-based co-activation networks, which were derived from ICA analyses on peak activation coordinates archived in a large neuroimaging database (i.e. BrainMap Database) [21]. On the basis of their spatial overlap with the templates, 6 ICs were identified as RSNs of interest. The DMN was divided into an anterior (aDMN) and a posterior component (pDMN) (Figure 1A and B) and the FPNs comprised of two lateralized components (i.e. right and left) (Figure 1C and D). The MPN and SN were each identified as a single component (Figure 1E and F). The highest voxel-wise spatial overlap between the 6 ICs and the corresponding templates were similar to previously reported values (r = 0.31–0.55; [49], [50]). The last step in the selection procedure was to examine the spectral characteristics of the 6 selected ICs using the same procedure as in Allen and colleagues [18]. For each group-level IC, we estimated the difference between the peak spectral power and minimum spectral power at frequencies to the right of the peak (i.e. dynamic range), and the ratio of the integral of power below 0.10 Hz to the integral of power between 0.15 and 0.25 Hz (i.e. low frequency to high frequency power ratio). Visual inspection of the scatter plot of low frequency to high frequency power ratio versus dynamic range confirmed that all 6 ICs are dominated by frequency fluctuations inside the 0.01–0.1 Hz window (Figure S1).

#### Within-network connectivity analysis

Statistical inference was carried out on the subject-specific *z*-maps of the 6 RSNs of interest using SPM8. For each group, we computed one-sample *t*-test to create statistical maps of the 6 components of interest (Figure 1). Threshold for the one-sample t-test was set at *p*<0.05 (Family-wise error, FWE correction) to select only the core nodes of each RSN. Subsequently, we created a binary representation of the conjunction of the statistical maps of the two groups, obtained from the one-sample *t* test results, which was further masked to exclude the non-gray matter voxels (Table S2). Group comparisons were restricted to voxels within these binary masks. Two-sample *t*-tests were performed to compare the 6 RSNs between the two groups (*p*<0.05 FWE correction). In addition, to account for type I error inflation for testing 6 RSNs, voxels were only considered significant if they survived a subsequent Bonferroni correction for multiple testing at p<0.008 (0.05/6). Age, gender and maximum motion were used as nuisance variables in the analysis.

#### Between-networks connectivity analysis

The subject-specific time courses of each RSN were entered in the FNC analysis (http://mialab.mrn.org/software). The FNC estimates the Pearson's correlation coefficient between pairs of time courses with a maximal lagged correlation approach (i.e. −3 to +3 s lag) [32]. Time course data were first band-pass filtered with a Butterworth filter with cutoff frequencies of.01–.1 Hz [51] and then pairwise correlations were computed between the time courses of the 6 RSNs of interest, resulting in 15 total pairwise correlations for each subject. Resulting Pearson's correlation coefficients were transformed to Fisher's z values and within-group correlations between networks as well as between-group differences in correlation coefficients were estimated. To control for multiple comparisons, p-values were thresholded according to a false discovery rate (FDR) of 0.05. Age gender and maximum motion were used as nuisance variables in the analysis.

#### Correlations with variables related to the clinical course of the disease

Additionally, we tested the effect of variables related to the clinical course of the disease on abnormal within- and between-networks functional connectivity. Using the MarsBaR toolbox (http://marsbar.sourceforge.net) we extracted the mean subject-specific *z*-values of the clusters showing significant between-group differences (p<.05, FDR correction) in the within–network connectivity and subsequently we conducted bivariate Pearson's correlations between the extracted values and the illness-related variables within the patient group. Similarly, bivariate Pearson's correlations were conducted between correlation coefficients estimating FNC between the MPN and right FPN (aberrant pair of networks) and variables related to the clinical course of the disease within the BD group.

To address the question whether the history of psychotic symptoms influences abnormal connectivity patterns, we divided the patient group into patients with (n = 13) and without (n = 17) history of psychotic symptoms and we performed a two-sample T-test to examine between-group differences in functional connectivity within- and between-networks (limited to aberrant connectivity).

## Results

### Components of interest and statistical comparison of RSNs

The 6 RSNs of interest were identified separately for the HC and BD group by performing one-sample-t-tests on the subject specific *z*-maps (*p*<0.05, FWE-corrected, Figure 1). Consistent with previous studies that used a high order model, the DMN was divided into an anterior part (aDMN) that covers the medial PFC/ACC (BA 10/32/24) and a posterior part (pDMN) that covers the precuneus/posterior cingulate cortex and angular gyri (BA 23/39) (Figure 1A and 1B). The right and left fronto-parietal networks mainly covered the inferior parietal cortex (BA 39/40) dorsolateral PFC (BA 45/9) and ventrolateral PFC (BA 46/47) (Figure 1C and 1D). The SN encompassed the anterior insula/lateral orbitofrontal cortex (BA 38/45/47) and the supramarginal gyrus (BA 40) (Figure 1E). The MPN included the amygdala, hippocampus, parahippocampal gyrus, temporal poles, and a part of insular cortex (BA 28/30/34/38) (Figure 1F). The location of the FC peaks and corresponding z-scores of every network are presented in Table S3.

There were no significant between-group differences within the 6 RSN of interest. An exploratory analysis without Bonferroni correction for testing multiple components also yielded no significant between-group differences.

### Between network connectivity

**Figure 2:**
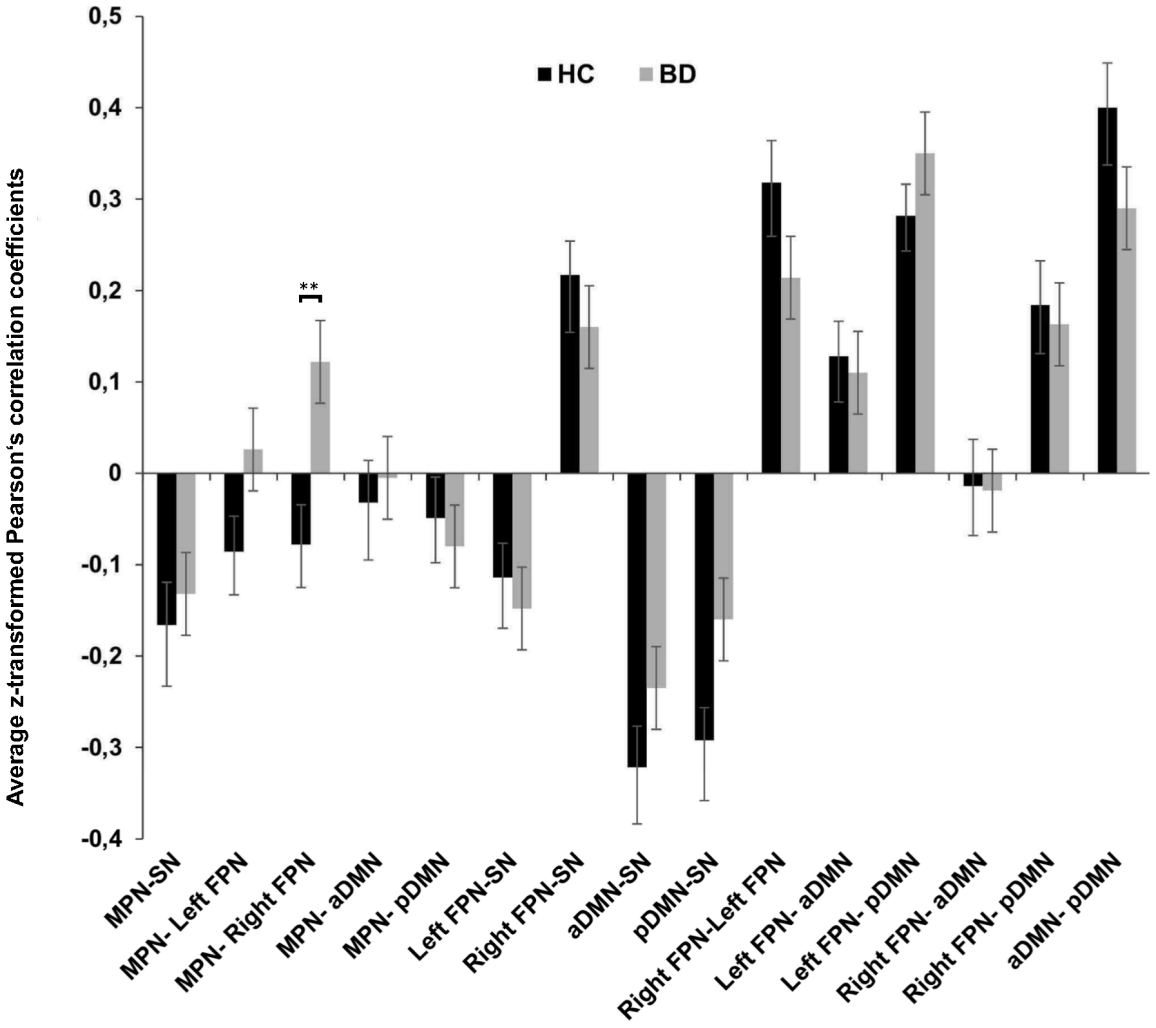
Functional network connectivity (FNC) between components. Average Fisher's z-transformed Pearson's correlation coefficients from FNC analysis reflecting connectivity between the 6 components of interest (15 possible pairs of components) for the healthy control (HC) and patient group (BD). Error bars represent the standard error of the mean (SEM). Asterisks (**) depict FDR significant finding.

**Figure 3:**
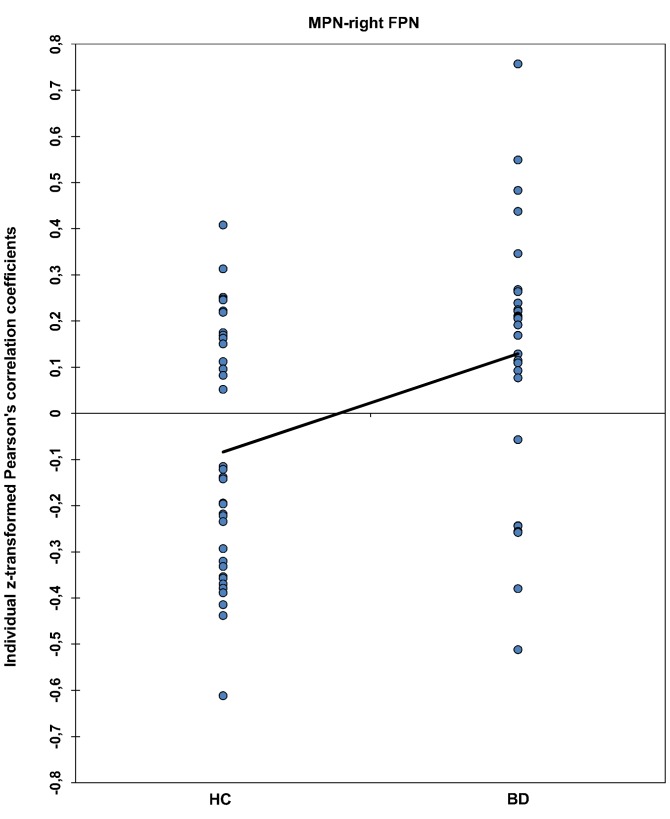
Functional network connectivity (FNC) between MPN-right FPN in the individual level. Fisher's z-transformed Pearson's correlation coefficients depicting individual FNC values for the MPN-right FPN pair. Both samples are normally distributed and have equal variance. The line connects the average FNC values of the two groups.

The 15 possible pairwise network combinations were tested for significant maximal-lagged correlations in each group (Figure 2 and Table 2). As expected, both groups showed strong positive connectivity between the right and left FPN and between the pDMN and aDMN. The SN showed positive FNC with the right FPN and negative FNC with the left FPN and the two DMNs. Both groups showed positive connectivity between the pDMN and the two lateralized fronto-parietal networks as well as between the aDMN and the left fronto-parietal network. We observed significant group differences in FNC between the MPN and the right FPN (p = .001, FDR-corrected; Figure 2 and 3). Whereas this combination of networks showed a tendency towards negative FNC in the HC group (r = −0.078), there was significant positive FNC between the two networks in the BD group (r = 0.122). Mean lag times did not differ between groups for any of the 15 combinations.

**Table 2:** Pearson's correlation coefficients and p values from FNC analysis.

Component combination	HC	BD	Between groups *p* (FDR corrected)
MPN-SN	−0.166*	−0.132*	0.835
MPN- Left FPN	−0.086	0.026	0.069
MPN- Right FPN	−0.078	0.122*	0.001§
MPN- aDMN	−0.032	−0.005	0.890
MPN- pDMN	−0.049	−0.080	0.794
Left FPN-SN	−0.114*	−0.148*	0.579
Right FPN-SN	0.217*	0.160*	0.335
aDMN-SN	−0.322*	−0.235*	0.219
pDMN-SN	−0.292*	−0.160	0.122
Right FPN-Left FPN	0.318*	0.214*	0.098
Left FPN- aDMN	0.128*	0.110*	0.829
Left FPN- pDMN	0.282*	0.350*	0.216
Right FPN- aDMN	−0.014	−0.019	0.838
Right FPN- pDMN	0.184*	0.163*	0.566
aDMN- pDMN	0.400*	0.290*	0.205

* Denotes significant (*p*<0.05 FDR corrected) Pearson's coefficient correlations between components within each group.

§ Denotes significant (*p*<0.05 FDR corrected) group differences in the magnitude of the correlation between components.

### Relationship with variables related to the clinical course of the disease

We conducted bivariate Pearson's correlations to investigate the relationship between abnormal functional connectivity and variables related to the clinical course of the disease or medication load within the BD group. There was no correlation between illness-related variables or medication load and abnormal between-networks connectivity patterns even when no correction for multiple testing was applied. Furthermore, the two-sample T-test revealed no differences in functional connectivity between patients with and without history of psychotic symptoms.

## Discussion

The present study extends previous knowledge by investigating functional connectivity within and between RSNs that have not been examined in detail despite their potential relevance for both affective and cognitive dysfunctions in BD. Compared to previous RS-fMRI studies in BD that employed an exploratory multivariate method, the present study comprised a larger sample of euthymic BD-I patients without current psychotic symptoms. Analysis of between-network connectivity revealed that in contrast to HC subjects where activity of the executive network does not correlate with activity of the MPN, BD patients display an increased interaction between the MPN and the right FPN.

To better interpret this finding, we drew upon evidence from meta-analytic studies that compared activation maps of different behavioral paradigms with ICA-derived spatial maps of RSNs [17], [21]. In this context, the positive FNC between the MPN and the right FPN in the BD group may reflect abnormal communication between a limbic-paralimbic network implicated in emotional processing and a right-lateralized dorsal network involved in executive functions and cognitive control [21]. In line with this assumption, neurobiological models of BD proposed that impaired emotion regulation in BD may result from a functional imbalance between a dorsal-cognitive network (i.e. dorsal ACC, dorsal PFC, inferior parietal cortex), and a ventral-emotional network (i.e. amygdala, parahippocampal gyrus, striatum, subgenual cingulate cortex, orbitofrontal cortex) [1], [4], [52]. In this respect, it is noteworthy that a trend-level group-related effect was also observed for the MPN-left FPN pair (p = .069) in the same direction as the main MPN-right FPN finding. Both findings suggest a general functional imbalance of the limbic network with fronto-parietal/cognitive networks in BD.

The present finding may be interpreted as impaired top-down control or abnormally increased bottom-up interference between these networks through the reciprocal connections between limbic-paralimbic structures and lateral PFC regions [53]. Independent of the direction of the interaction (i.e. top-down or bottom-up), this result supports previous evidence from seed-based studies showing abnormal FC between limbic structures, such as amygdala, and right PFC at rest [13], [16] or during emotional tasks [9], [54], [55]. Our results are also in line with a recent meta-analysis showing increased ventral-limbic activation and decreased right-lateralized PFC activation during emotional processing in BD patients [37].

Aberrant FNC between the MPN and the right FPN in the BD group did not correlate with medication load or variables characterizing the clinical course of the disease, namely, the number of manic and depressive episodes, the age of illness onset, the time in remission, and the history of psychotic symptoms. Previous research has shown that some of these clinical characteristics of BD patients correlate with behavioral variables [38], [39] and with brain activation and connectivity patterns [5], [13]. The absence of significant correlations in the present sample of euthymic BD patients with minimum residual mood symptoms and no current psychotic symptoms indicates that aberrant FNC between the MPN and the right FPN may constitute a trait marker of BD. Emerging questions, for future studies to address, focus on whether this FC abnormality reflects a developmental failure to establish healthy prefrontal–limbic modulation early in life which may later result in the onset of the disease [5] and may be present in healthy populations at high risk for developing BD or represents common state- and mood-independent effects of previous BD episodes.

Contrary to our hypothesis, we did not observe abnormal within-network FC in any of the 6 RSNs of interest also when no correction for multiple comparisons was applied. In line with the lack of significant within-network FC findings in the present study, a recent RS-fMRI study showed significant BD-related impairments in between- but not in within-network FC [35]. This evidence leads to the assumption that BD like schizophrenia may be mainly characterized by dysconnectivity in large-scale networks [56], [57].

Based on our hypotheses, abnormal FC within and between the RSNs of interest may underlie affective and cognitive symptoms of the disorder. Therefore, these abnormalities would more likely characterize symptomatic rather than euthymic patients. In line with this idea, previous ICA studies that investigated functional connectivity patterns in BD at rest reported altered connectivity within the DMN in manic [34] or psychotic [22], [23] BD patients. The assumption that these alterations are state-dependent is further supported by evidence that these abnormal FC patterns are either shared by schizophrenic patients [22] or correlate with negative mood symptoms [23].

Another plausible explanation for the lack of BD-related abnormal FC in the present study may be the context-dependent nature of these abnormalities, which may only be present or more pronounced during cognitive and emotional tasks that engage these networks and not at rest. Future ICA studies comparing rest and task conditions should investigate this possibility.

The present findings have to be interpreted in the light of some limitations. First, inherent to the nature of the ICA, there is no optimal way to estimate the number of ICs (problem of dimensionality). Yet, the number of ICs has a significant impact on the spatial characteristics of the RSNs [58]. Most studies use the Minimum Description Length criteria as standard criteria to determine the number of ICs. However, using these criteria, we were not able to identify all common RSNs that were of interest for the present study. As previous studies showed that detection of some RSNs requires higher dimensionality models [44], we decided to gradually increase the dimensionality until the point that all the networks of interest were identified [44]. The aim was to find the minimum number of components in which our hypotheses could be tested. Additionally, we performed the same analysis with a standard high model order ICA (i.e. 75 ICs), as previous studies have demonstrated that this model order yield refined components that correspond to known anatomical and functional segmentations [17], [44], [45]. Importantly, we found similar results concerning the MPN with both 40 and 75 ICs (see Analysis S1).

A second limitation is related to the correlation analysis between abnormal FC patterns and clinical characteristics. The absence of correlations may also be related to the lack of power as the sample was relatively small for this type of analysis and patients investigated here were relatively healthy leading to little variance with regard to symptomatic variables.

Furthermore, medication load was assessed as a composite measure assuming synergistic rather than antagonistic effects of the different types of medication namely antidepressants, mood stabilizers, and antipsychotics. It is rather unlikely that the effects of different classes of medication on brain connectivity are synergistic and independent from each other. Therefore, category-specific medication effects may still confound the present findings. However, due to possible pharmacological interactions between different types of medication the independent impact of every class of medication on connectivity cannot be directly assessed in the present sample. Future studies using medication-based selection criteria for the patient group and a drug-free baseline are more suitable to address questions about potential pharmacological effects on brain connectivity.

Finally, the interpretation of resting state data in light of previous task-related neuroimaging studies has to be made with caution. It still remains an open question to what extent task-based differences in specific brain regions in BD are reflected in different connectivity patterns of their respective brain networks at rest. Furthermore, to date, there is very sparse evidence linking RSNs with behavior and cognitive or emotional processes which could provide functional interpretations of widely observed RSNs.

In conclusion, the present study substantially extends prior work in BD by employing a data-driven multivariate approach to examine euthymic BD-I patients without current psychotic symptoms. We showed abnormal interactions between the MPN and the right FPN in BD at rest. This abnormality may underlie impaired integration of affective and cognitive processes leading to dysfunctional emotion regulation. Furthermore, the present results highlight the importance of the MPN in this disorder.

## Supporting Information

Figure S1 **Scatter plot of low frequency (LF) to high frequency (HF) power ratio versus dynamic range for all components.** Red squares represent the 6 components of interest selected in the present study.
(TIF)

Table S1 **Voxel-wise spatial overlap (Pearson's r) of RSNs of interest with gray matter, white matter, cerebrospinal fluid masks and templates of RSNs.**
(XLSX)

Table S2 **Number of voxels before and after excluding non-gray matter voxels.**
(XLSX)

Table S3 **Peak within-network functional connectivity for both groups.**
(XLSX)

Analysis S1 **Group Independent component analysis with 75 ICs.**
(DOCX)

## References

[1] Phillips ML, Ladouceur CD, Drevets WC (2008) A neural model of voluntary and automatic emotion regulation: implications for understanding the pathophysiology and neurodevelopment of bipolar disorder. Mol Psychiatry 13: 829–857, 829, 833-857 18574483 10.1038/mp.2008.65 PMC2745893

[2] Mann-Wrobel MC, Carreno JT, Dickinson D (2011) Meta-analysis of neuropsychological functioning in euthymic bipolar disorder: an update and investigation of moderator variables. Bipolar Disord 13: 334–342. 21843273 10.1111/j.1399-5618.2011.00935.x

[3] Sole B, Bonnin CM, Torrent C, Martinez-Aran A, Popovic D, et al (2012) Neurocognitive impairment across the bipolar spectrum. CNS Neurosci Ther 18: 194–200. 22128808 10.1111/j.1755-5949.2011.00262.x PMC6493522

[4] Phillips ML, Drevets WC, Rauch SL, Lane R (2003) Neurobiology of emotion perception II: Implications for major psychiatric disorders. Biol Psychiatry 54: 515–528. 12946880 10.1016/s0006-3223(03)00171-9

[5] Strakowski SM, Adler CM, Almeida J, Altshuler LL, Blumberg HP, et al (2012) The functional neuroanatomy of bipolar disorder: a consensus model. Bipolar Disord 14: 313–325. 22631617 10.1111/j.1399-5618.2012.01022.x PMC3874804

[6] Wessa M, Kanske P, Linke J (2013) Bipolar disorder: A neural network perspective on a disorder of emotion and motivation. Restor Neurol Neurosci 10.3233/RNN-139007 23603441

[7] Wessa M, Linke J (2009) Emotional processing in bipolar disorder: behavioural and neuroimaging findings. Int Rev Psychiatry 21: 357–367. 20374149 10.1080/09540260902962156

[8] Pompei F, Dima D, Rubia K, Kumari V, Frangou S (2011) Dissociable functional connectivity changes during the Stroop task relating to risk, resilience and disease expression in bipolar disorder. Neuroimage 57: 576–582. 21570470 10.1016/j.neuroimage.2011.04.055

[9] Foland LC, Altshuler LL, Bookheimer SY, Eisenberger N, Townsend J, et al (2008) Evidence for deficient modulation of amygdala response by prefrontal cortex in bipolar mania. Psychiatry Res 162: 27–37. 18063349 10.1016/j.pscychresns.2007.04.007 PMC2410029

[10] Townsend JD, Torrisi SJ, Lieberman MD, Sugar CA, Bookheimer SY, et al (2013) Frontal-amygdala connectivity alterations during emotion downregulation in bipolar I disorder. Biol Psychiatry 73: 127–135. 22858151 10.1016/j.biopsych.2012.06.030 PMC3525751

[11] Versace A, Thompson WK, Zhou D, Almeida JR, Hassel S, et al (2010) Abnormal left and right amygdala-orbitofrontal cortical functional connectivity to emotional faces: state versus trait vulnerability markers of depression in bipolar disorder. Biol Psychiatry 67: 422–431. 20159144 10.1016/j.biopsych.2009.11.025 PMC2835157

[12] Anand A, Li Y, Wang Y, Lowe MJ, Dzemidzic M (2009) Resting state corticolimbic connectivity abnormalities in unmedicated bipolar disorder and unipolar depression. Psychiatry Res 171: 189–198. 19230623 10.1016/j.pscychresns.2008.03.012 PMC3001251

[13] Anticevic A, Brumbaugh MS, Winkler AM, Lombardo LE, Barrett J, et al (2013) Global prefrontal and fronto-amygdala dysconnectivity in bipolar I disorder with psychosis history. Biol Psychiatry 73: 565–573. 22980587 10.1016/j.biopsych.2012.07.031 PMC3549314

[14] Chai XJ, Whitfield-Gabrieli S, Shinn AK, Gabrieli JD, Nieto Castanon A, et al (2011) Abnormal medial prefrontal cortex resting-state connectivity in bipolar disorder and schizophrenia. Neuropsychopharmacology 36: 2009–2017. 21654735 10.1038/npp.2011.88 PMC3158318

[15] Mamah D, Barch DM, Repovs G (2013) Resting state functional connectivity of five neural networks in bipolar disorder and schizophrenia. J Affect Disord 10.1016/j.jad.2013.01.051 PMC3749249 23489402

[16] Torrisi S, Moody TD, Vizueta N, Thomason ME, Monti MM, et al (2013) Differences in resting corticolimbic functional connectivity in bipolar I euthymia. Bipolar Disord 15: 156–166. 23347587 10.1111/bdi.12047 PMC3582748

[17] Smith SM, Fox PT, Miller KL, Glahn DC, Fox PM, et al (2009) Correspondence of the brain's functional architecture during activation and rest. Proc Natl Acad Sci U S A 106: 13040–13045. 19620724 10.1073/pnas.0905267106 PMC2722273

[18] Allen EA, Erhardt EB, Damaraju E, Gruner W, Segall JM, et al (2011) A baseline for the multivariate comparison of resting-state networks. Front Syst Neurosci 5: 2. 21442040 10.3389/fnsys.2011.00002 PMC3051178

[19] Damoiseaux JS, Rombouts SA, Barkhof F, Scheltens P, Stam CJ, et al (2006) Consistent resting-state networks across healthy subjects. Proc Natl Acad Sci U S A 103: 13848–13853. 16945915 10.1073/pnas.0601417103 PMC1564249

[20] Greicius MD, Supekar K, Menon V, Dougherty RF (2009) Resting-state functional connectivity reflects structural connectivity in the default mode network. Cereb Cortex 19: 72–78. 18403396 10.1093/cercor/bhn059 PMC2605172

[21] Laird AR, Fox PM, Eickhoff SB, Turner JA, Ray KL, et al (2011) Behavioral interpretations of intrinsic connectivity networks. J Cogn Neurosci 23: 4022–4037. 21671731 10.1162/jocn_a_00077 PMC3690655

[22] Khadka S, Meda SA, Stevens MC, Glahn DC, Calhoun VD, et al (2013) Is Aberrant Functional Connectivity A Psychosis Endophenotype? A Resting State Functional Magnetic Resonance Imaging Study. Biol Psychiatry 10.1016/j.biopsych.2013.04.024 PMC3752322 23746539

[23] Meda SA, Gill A, Stevens MC, Lorenzoni RP, Glahn DC, et al (2012) Differences in resting-state functional magnetic resonance imaging functional network connectivity between schizophrenia and psychotic bipolar probands and their unaffected first-degree relatives. Biol Psychiatry 71: 881–889. 22401986 10.1016/j.biopsych.2012.01.025 PMC3968680

[24] Damoiseaux JS, Beckmann CF, Arigita EJ, Barkhof F, Scheltens P, et al (2008) Reduced resting-state brain activity in the “default network” in normal aging. Cereb Cortex 18: 1856–1864. 18063564 10.1093/cercor/bhm207

[25] Corbetta M, Shulman GL (2002) Control of goal-directed and stimulus-driven attention in the brain. Nat Rev Neurosci 3: 201–215. 11994752 10.1038/nrn755

[26] Vincent JL, Kahn I, Snyder AZ, Raichle ME, Buckner RL (2008) Evidence for a frontoparietal control system revealed by intrinsic functional connectivity. J Neurophysiol 100: 3328–3342. 18799601 10.1152/jn.90355.2008 PMC2604839

[27] Menon V (2011) Large-scale brain networks and psychopathology: a unifying triple network model. Trends Cogn Sci 15: 483–506. 21908230 10.1016/j.tics.2011.08.003

[28] Seeley WW, Menon V, Schatzberg AF, Keller J, Glover GH, et al (2007) Dissociable intrinsic connectivity networks for salience processing and executive control. J Neurosci 27: 2349–2356. 17329432 10.1523/JNEUROSCI.5587-06.2007 PMC2680293

[29] Greicius MD (2003) Neuroimaging in developmental disorders. Curr Opin Neurol 16: 143–146. 12644740 10.1097/01.wco.0000063763.15877.d2

[30] Raichle ME, MacLeod AM, Snyder AZ, Powers WJ, Gusnard DA, et al (2001) A default mode of brain function. Proc Natl Acad Sci U S A 98: 676–682. 11209064 10.1073/pnas.98.2.676 PMC14647

[31] Sridharan D, Levitin DJ, Menon V (2008) A critical role for the right fronto-insular cortex in switching between central-executive and default-mode networks. Proc Natl Acad Sci U S A 105: 12569–12574. 18723676 10.1073/pnas.0800005105 PMC2527952

[32] Jafri MJ, Pearlson GD, Stevens M, Calhoun VD (2008) A method for functional network connectivity among spatially independent resting-state components in schizophrenia. Neuroimage 39: 1666–1681. 18082428 10.1016/j.neuroimage.2007.11.001 PMC3164840

[33] Chepenik LG, Raffo M, Hampson M, Lacadie C, Wang F, et al (2010) Functional connectivity between ventral prefrontal cortex and amygdala at low frequency in the resting state in bipolar disorder. Psychiatry Res 182: 207–210. 20493671 10.1016/j.pscychresns.2010.04.002 PMC2914819

[34] Ongur D, Lundy M, Greenhouse I, Shinn AK, Menon V, et al (2010) Default mode network abnormalities in bipolar disorder and schizophrenia. Psychiatry Res 183: 59–68. 20553873 10.1016/j.pscychresns.2010.04.008 PMC2902695

[35] Das P, Calhoun V, Malhi GS (2014) Bipolar and borderline patients display differential patterns of functional connectivity among resting state networks. Neuroimage 10.1016/j.neuroimage.2014.04.062 24793833

[36] Chen CH, Suckling J, Lennox BR, Ooi C, Bullmore ET (2011) A quantitative meta-analysis of fMRI studies in bipolar disorder. Bipolar Disord 13: 1–15. 10.1111/j.1399-5618.2011.00893.x 21320248

[37] Houenou J, Frommberger J, Carde S, Glasbrenner M, Diener C, et al (2011) Neuroimaging-based markers of bipolar disorder: evidence from two meta-analyses. J Affect Disord 132: 344–355. 21470688 10.1016/j.jad.2011.03.016

[38] Linke J, Sonnekes C, Wessa M (2011) Sensitivity to positive and negative feedback in euthymic patients with bipolar I disorder: the last episode makes the difference. Bipolar Disord 13: 638–650. 22085477 10.1111/j.1399-5618.2011.00956.x

[39] Wright KA, Lam D, Brown RG (2008) Dysregulation of the behavioral activation system in remitted bipolar I disorder. J Abnorm Psychol 117: 838–848. 19025230 10.1037/a0013598

[40] Sackeim HA (2001) The definition and meaning of treatment-resistant depression. J Clin Psychiatry 62 Suppl 16: 10–17. 11480879

[41] Gardner DM, Murphy AL, O'Donnell H, Centorrino F, Baldessarini RJ (2010) International consensus study of antipsychotic dosing. Am J Psychiatry 167: 686–693. 20360319 10.1176/appi.ajp.2009.09060802

[42] Van Dijk KR, Sabuncu MR, Buckner RL (2012) The influence of head motion on intrinsic functional connectivity MRI. Neuroimage 59: 431–438. 21810475 10.1016/j.neuroimage.2011.07.044 PMC3683830

[43] Calhoun VD, Adali T, Pearlson GD, Pekar JJ (2001) A method for making group inferences from functional MRI data using independent component analysis. Hum Brain Mapp 14: 140–151. 11559959 10.1002/hbm.1048 PMC6871952

[44] Abou-Elseoud A, Starck T, Remes J, Nikkinen J, Tervonen O, et al (2010) The effect of model order selection in group PICA. Hum Brain Mapp 31: 1207–1216. 20063361 10.1002/hbm.20929 PMC6871136

[45] Kiviniemi V, Starck T, Remes J, Long X, Nikkinen J, et al (2009) Functional segmentation of the brain cortex using high model order group PICA. Hum Brain Mapp 30: 3865–3886. 19507160 10.1002/hbm.20813 PMC6870574

[46] Bell AJ, Sejnowski TJ (1995) An information-maximization approach to blind separation and blind deconvolution. Neural Comput 7: 1129–1159. 7584893 10.1162/neco.1995.7.6.1129

[47] Himberg J, Hyvarinen A, Esposito F (2004) Validating the independent components of neuroimaging time series via clustering and visualization. Neuroimage 22: 1214–1222. 15219593 10.1016/j.neuroimage.2004.03.027

[48] Erhardt EB, Rachakonda S, Bedrick EJ, Allen EA, Adali T, et al (2011) Comparison of multi-subject ICA methods for analysis of fMRI data. Hum Brain Mapp 32: 2075–2095. 21162045 10.1002/hbm.21170 PMC3117074

[49] Assaf M, Jagannathan K, Calhoun VD, Miller L, Stevens MC, et al (2010) Abnormal functional connectivity of default mode sub-networks in autism spectrum disorder patients. Neuroimage 53: 247–256. 20621638 10.1016/j.neuroimage.2010.05.067 PMC3058935

[50] von dem Hagen EA, Stoyanova RS, Baron-Cohen S, Calder AJ (2013) Reduced functional connectivity within and between ‘social’ resting state networks in autism spectrum conditions. Soc Cogn Affect Neurosci 8: 694–701. 22563003 10.1093/scan/nss053 PMC3739917

[51] Cordes D, Haughton VM, Arfanakis K, Carew JD, Turski PA, et al (2001) Frequencies contributing to functional connectivity in the cerebral cortex in “resting-state” data. AJNR Am J Neuroradiol 22: 1326–1333. 11498421 PMC7975218

[52] Strakowski SM, Adler CM, Holland SK, Mills NP, DelBello MP, et al (2005) Abnormal FMRI brain activation in euthymic bipolar disorder patients during a counting Stroop interference task. Am J Psychiatry 162: 1697–1705. 16135630 10.1176/appi.ajp.162.9.1697

[53] Mayberg HS, Liotti M, Brannan SK, McGinnis S, Mahurin RK, et al (1999) Reciprocal limbic-cortical function and negative mood: converging PET findings in depression and normal sadness. Am J Psychiatry 156: 675–682. 10327898 10.1176/ajp.156.5.675

[54] Perlman SB, Almeida JR, Kronhaus DM, Versace A, Labarbara EJ, et al (2012) Amygdala activity and prefrontal cortex-amygdala effective connectivity to emerging emotional faces distinguish remitted and depressed mood states in bipolar disorder. Bipolar Disord 14: 162–174. 22420592 10.1111/j.1399-5618.2012.00999.x PMC3703524

[55] Vizueta N, Rudie JD, Townsend JD, Torrisi S, Moody TD, et al (2012) Regional fMRI hypoactivation and altered functional connectivity during emotion processing in nonmedicated depressed patients with bipolar II disorder. Am J Psychiatry 169: 831–840. 22773540 10.1176/appi.ajp.2012.11030349 PMC3740182

[56] Uhlhaas PJ, Singer W (2012) Neuronal dynamics and neuropsychiatric disorders: toward a translational paradigm for dysfunctional large-scale networks. Neuron 75: 963–980. 22998866 10.1016/j.neuron.2012.09.004

[57] Uhlhaas PJ (2013) Dysconnectivity, large-scale networks and neuronal dynamics in schizophrenia. Curr Opin Neurobiol 23: 283–290. 23228430 10.1016/j.conb.2012.11.004

[58] Cole DM, Smith SM, Beckmann CF (2010) Advances and pitfalls in the analysis and interpretation of resting-state FMRI data. Front Syst Neurosci 4: 8. 20407579 10.3389/fnsys.2010.00008 PMC2854531

