# Supplementary figures and images for: Altered Functional Connectivity between Emotional and Cognitive Resting State Networks in Euthymic Bipolar I Disorder Patients
Source: PLoS One. 2014 Oct 24;9(10):e107829. doi: 10.1371/journal.pone.0107829 (PMC4208743; doi:10.1371/journal.pone.0107829)

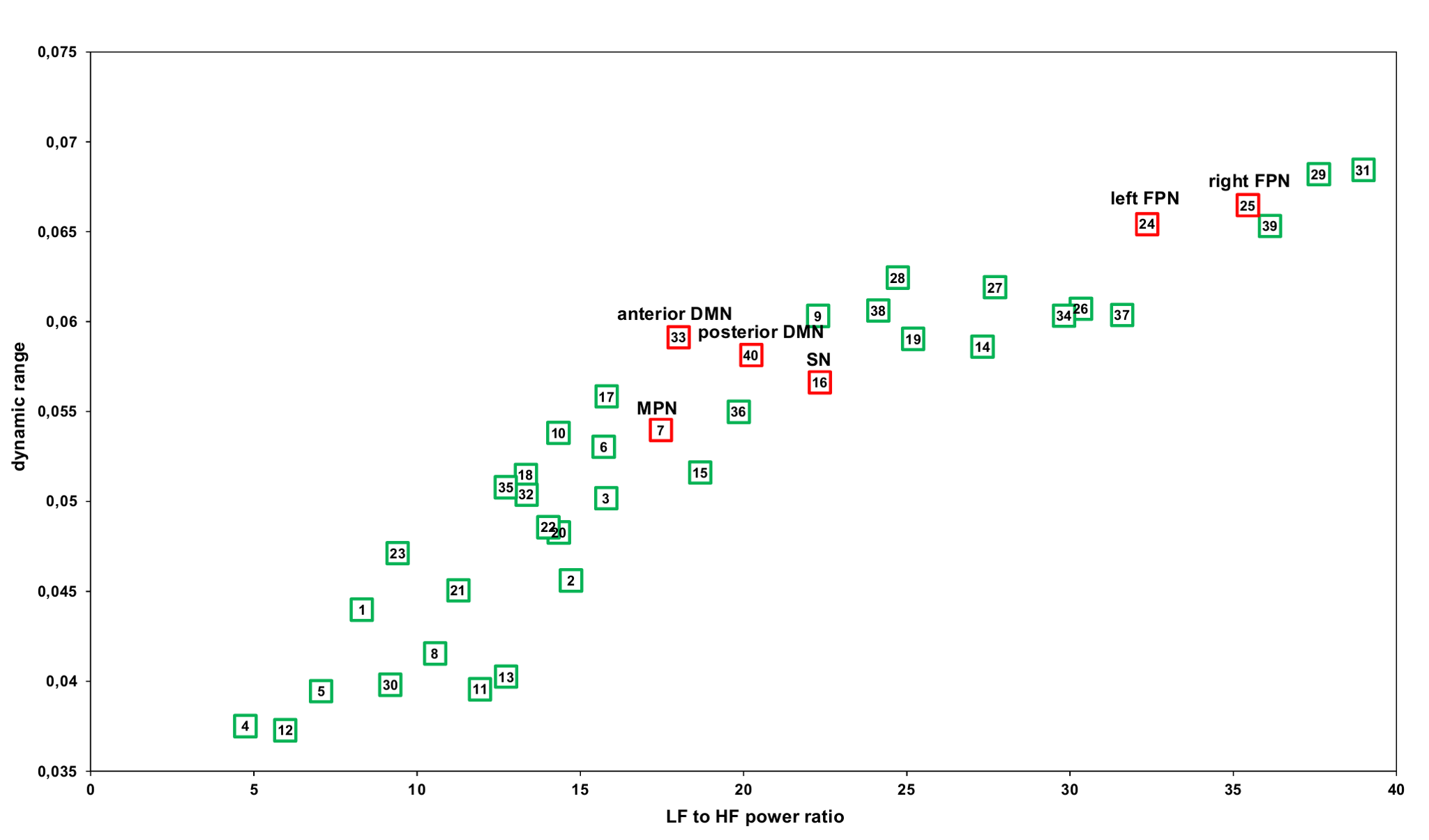

Supplement: Figure S1 — Scatter plot of low frequency (LF) to high frequency (HF) power ratio versus dynamic range for all components. Red squares represent the 6 components of interest selected in the present study. (TIF) [file pone.0107829.s001.tif]
