# Supplementary material for: Altered Functional Connectivity between Emotional and Cognitive Resting State Networks in Euthymic Bipolar I Disorder Patients
Source: PLoS One. 2014 Oct 24;9(10):e107829. doi: 10.1371/journal.pone.0107829 (PMC4208743; doi:10.1371/journal.pone.0107829)
Supplement: Analysis S1 — Group Independent component analysis with 75 ICs. (DOCX) [file pone.0107829.s005.docx]

**Analysis S1**

**A. Group Independent component analysis with 75 ICs**

Using the same parameters as in the main analysis, we performed a Group-ICA analysis with 75 ICs components. Based on previous ICA studies, this high model order yield refined components that correspond to known anatomical and functional segmentation [[1-3](#_ENREF_1)]. Similar to the lower model order analysis (i.e. 40ICs), six RSNs of interest (i.e. aDMN, pDMN, right FPN, left FPN, SN, and MPN) were identified in both the HC and BD group by testing the main effect of group on the subject specific *z*-maps of these networks (*p* < 0.05, FWE-corrected). The spatial maps of the six components of interest were similar to the networks identified with the lower model order.

We observed between-group differences in the voxel-wise spatial distribution of the functional connectivity maps in the MPN network (local FDR-corrected at *p* < 0.05) (Table 1 main text). As in the lower model order analysis (i.e. 40ICs), increased engagement of the left parahippocampal gyrus within the MPN was revealed in the BD compared to HC group.

| **Brain region** | **Brodmann area** | **MNI Coordinates** | | | **Cluster size** | **Z** | **p_FDR-corrected_** |
| --- | --- | --- | --- | --- | --- | --- | --- |
|  |  | **x** | **y** | **z** |  |  |  |
| **BD > HC** |  |  |  |  |  |  |  |
| Parahippocampal Gyrus (Left) | 28 | -20 | 3 | -25 | 4 | 4.04 | .039 |

**B. Between networks connections**

The 15 possible pairwise network combinations were also tested for significant maximal-lagged between-networks connectivity. We observed a similar FNC pattern for both HC and BD group as in the low model order. Most importantly, the MPN-right FPN pair showed a trend toward increased FNC between-group differences (p = .054) in the BD group.

| **Component combination** | **HC** | **BD** | **Between groups**  ***p*_FDR-corrected_** |
| --- | --- | --- | --- |
| MPN- Right FPN | 0,055 | 0,185* | 0,054 |
| MPN- Left FPN | 0,035 | 0,122* | 0,134 |
| MPN- aDMN | 0,134* | 0,249* | 0,194 |
| MPN- pDMN | -0,082 | -0,082 | 0,997 |
| MPN-SN | -0,202* | -0,236* | 0,738 |
| Right FPN-Left FPN | 0,431* | 0,410* | 0,742 |
| Right FPN- aDMN | 0,219* | 0,252* | 0,651 |
| Right FPN- pDMN | 0,220* | 0,162* | 0,476 |
| Right FPN-SN | 0,184* | 0,099 | 0,260 |
| Left FPN- aDMN | 0,378* | 0,364* | 0,786 |
| Left FPN- pDMN | 0,393* | 0,370* | 0,733 |
| Left FPN-SN | -0,156* | -0,166* | 0,880 |
| aDMN- pDMN | 0,308* | 0,215* | 0,270 |
| aDMN-SN | -0,116 | -0,189* | 0,433 |
| pDMN-SN | -0,029 | 0,060 | 0,368 |

* Denotes significant (*p* < 0.05 FDR corrected) Pearson’s coefficient correlations between components within each group.

**References**

1. Abou-Elseoud A, Starck T, Remes J, Nikkinen J, Tervonen O, et al. (2010) The effect of model order selection in group PICA. Hum Brain Mapp 31: 1207-1216.

2. Kiviniemi V, Starck T, Remes J, Long X, Nikkinen J, et al. (2009) Functional segmentation of the brain cortex using high model order group PICA. Hum Brain Mapp 30: 3865-3886.

3. Smith SM, Fox PT, Miller KL, Glahn DC, Fox PM, et al. (2009) Correspondence of the brain's functional architecture during activation and rest. Proc Natl Acad Sci U S A 106: 13040-13045.
